# Testing the Efficacy of a Multicomponent, Self-Guided, Smartphone-Based Meditation App: Three-Armed Randomized Controlled Trial

**DOI:** 10.2196/23825

**Published:** 2020-11-27

**Authors:** Simon B Goldberg, Theodore Imhoff-Smith, Daniel M Bolt, Christine D Wilson-Mendenhall, Cortland J Dahl, Richard J Davidson, Melissa A Rosenkranz

**Affiliations:** 1 Center for Healthy Minds University of Wisconsin Madison, WI United States; 2 Department of Counseling Psychology University of Wisconsin-Madison Madison, WI United States; 3 Department of Educational Psychology University of Wisconsin-Madison Madison, WI United States; 4 Healthy Minds Innovations Inc Madison, WI United States; 5 Department of Psychology University of Wisconsin-Madison Madison, WI United States; 6 Department of Psychiatry University of Wisconsin-Madison Madison, WI United States

**Keywords:** meditation, mindfulness, compassion, mobile health, loneliness, randomized controlled trial, mobile phone

## Abstract

**Background:**

A growing number of randomized controlled trials (RCTs) suggest psychological benefits associated with meditation training delivered via mobile health. However, research in this area has primarily focused on mindfulness, only one of many meditative techniques.

**Objective:**

This study aims to evaluate the efficacy of 2 versions of a self-guided, smartphone-based meditation app—the Healthy Minds Program (HMP)—which includes training in mindfulness (Awareness), along with practices designed to cultivate positive relationships (Connection) or insight into the nature of the self (Insight).

**Methods:**

A three-arm, fully remote RCT compared 8 weeks of one of 2 HMP conditions (Awareness+Connection and Awareness+Insight) with a waitlist control. Adults (≥18 years) without extensive previous meditation experience were eligible. The primary outcome was psychological distress (depression, anxiety, and stress). Secondary outcomes were social connection, empathy, compassion, self-reflection, insight, rumination, defusion, and mindfulness. Measures were completed at pretest, midtreatment, and posttest between October 2019 and April 2020. Longitudinal data were analyzed using intention-to-treat principles with maximum likelihood.

**Results:**

A total of 343 participants were randomized and 186 (54.2%) completed at least one posttest assessment. The majority (166/228, 72.8%) of those assigned to HMP conditions downloaded the app. The 2 HMP conditions did not differ from one another in terms of changes in any outcome. Relative to the waitlist control, the HMP conditions showed larger improvements in distress, social connectedness, mindfulness, and measures theoretically linked to insight training (*d*=–0.28 to 0.41; *P*s≤.02), despite modest exposure to connection- and insight-related practice. The results were robust to some assumptions about nonrandom patterns of missing data. Improvements in distress were associated with days of use. Candidate mediators (social connection, insight, rumination, defusion, and mindfulness) and moderators (baseline rumination, defusion, and empathy) of changes in distress were identified.

**Conclusions:**

This study provides initial evidence of efficacy for the HMP app in reducing distress and improving outcomes related to well-being, including social connectedness. Future studies should attempt to increase study retention and user engagement.

**Trial Registration:**

ClinicalTrials.gov NCT04139005; https://clinicaltrials.gov/ct2/show/NCT04139005

## Introduction

### Background

Mindfulness and meditation have become household words for many people in the United States and across the globe in the past 20 years. Derived from Buddhist and Hindu contemplative traditions [1], secularized meditative practices are being taught in schools, recommended by health care providers, and employed by businesses [2-4]. The use of meditation tripled in the United States between 2012 and 2017 (from 4.1% to 14.2%) [5]. Meta-analyses involving hundreds of randomized controlled trials (RCTs) suggest that meditation training can decrease psychological symptoms (eg, depression, anxiety, stress) and increase aspects of well-being and positive functioning (eg, meaning in life, compassion, prosocial behavior) [6-15].

To date, the vast majority of research on meditation has focused on interventions delivered in person. Standardized mindfulness-based interventions such as mindfulness-based stress reduction (MBSR) [16] and mindfulness-based cognitive therapy (MBCT) [17] were explicitly designed as group-based interventions delivered by trained instructors, and these interpersonal elements are viewed as central ingredients (eg, group format) [18]. Despite some meditation-based interventions (MBIs) being recommended as first-line treatments (eg, MBCT for depression relapse prevention) [19-21], their availability remains limited [22]. Barriers for the dissemination of MBIs are similar to those facing other evidence-based psychotherapies (eg, lack of available providers, cost, logistical challenges) [23-25].

Delivering interventions through mobile technology has been proposed as a solution for increasing access to psychological interventions, including MBIs [26,27]. Web- and smartphone-based interventions have obvious advantages over traditional in-person delivery in terms of cost and scalability. Furthermore, mobile health (mHealth) interventions can, in theory, do things that in-person interventions typically never do, such as providing access 24 hours a day or customizing content based on passively sensed data (eg, location) [28]. Among mHealth delivery platforms, smartphone-based interventions may be particularly promising, with these devices often kept within arm’s reach, charged, turned on, and being owned by the vast majority of the population [29].

There has been a dramatic increase in the past five years in RCTs testing smartphone-based interventions that include training in meditation [30]. These studies have begun to examine efficacy in various clinical and nonclinical populations [31-38]. Although preliminary, available evidence suggests that smartphone-based interventions that include training in meditation and mindfulness may provide psychological benefits that are similar to in-person MBIs (eg, decreased psychological symptoms, increased positive functioning), albeit smaller in magnitude [30,39-42].

Similar to the in-person MBI literature, RCTs testing the mobile delivery of MBIs have focused almost entirely on mindfulness. The term *mindfulness* is derived from the Pali word *sati*, which in Buddhism refers to the cultivation of receptive, present-moment awareness [43]. In the scientific literature, mindfulness can refer to a mental state, trait, or faculty amenable to training [44-47]. Mindfulness-based interventions commonly adopt the definition by Kabat-Zinn [48]: “paying attention in a particular way: on purpose, in the present moment, and nonjudgmentally.” Meta-analyses suggest that dispositional mindfulness along with both short-term (eg, mindfulness inductions) and long-term (eg, mindfulness-based interventions such as MBSR) training are associated with decreased psychiatric symptoms, negative affect, substance use, and neuroticism [9,49-53].

Importantly, mindfulness training represents the implementation of primarily one meditative approach drawn from rich contemplative traditions [54]. Although largely untested, it is possible that a variety of meditative techniques may serve as valuable complements or alternatives to mindfulness. Different meditation practices have shown distinct neural signatures [55,56] and can produce different psychological effects [57]. Dahl et al [54] provide a useful typology for situating mindfulness training within the broader contemplative practice landscape. Using a family resemblance approach, they describe *attentional*, *constructive*, and *deconstructive* families. Mindfulness meditation, as implemented in MBSR, falls primarily within the *attentional family*, with training focused on regulating attention. The *constructive family* includes practices designed to strengthen psychological habits conducive to psychosocial health. This includes connection-based practices that involve cultivating feelings of warmth and friendliness toward oneself and others (eg, gratitude, loving kindness, and compassion practices) [58,59]. Experimental evidence suggests that connection practices increase well-being and decrease psychological symptoms [8,60]. The *deconstructive family* includes practices designed to modify unhelpful cognitive patterns, particularly regarding one’s view of self and others. Practices in this family involve intentional self-inquiry into the dynamics of conscious experience and the nature of the self with the goal of generating an understanding of cognitive patterns (ie, insight). Deconstructive elements are present in MBCT and cognitive therapy more generally (eg, seeing thoughts as thoughts) [17,61]. However, research on deconstructive meditative practices has been limited.

Smartphone-based meditation interventions have almost exclusively focused on mindfulness training [30,62], although several studies have investigated internet-based interventions that include connection-related practices [63-65]. Although some interventions include connection-based practice as one of several guided practices within a general mindfulness framework [32], RCTs primarily examining constructive or deconstructive practices are rare (with some promising exceptions) [66,67].

### This Study

This study sought to investigate the effects of a self-guided, smartphone-based meditation intervention that included explicit training in constructive and deconstructive families of practices [54]. In a three-arm RCT, we compared training in mindful awareness, paired with Connection or Insight practices, with a waitlist control. As both arms included the Awareness module first, we refer to them by their unique module (ie, Connection or Insight, rather than Awareness+Connection and Awareness+Insight). We included outcome measures designed to detect global effects (psychological distress) and practice-specific effects (eg, social connection, shift in relationship to one’s thoughts). Our primary hypothesis was that participants in both active conditions would show reduced psychological distress relative to the waitlist control. In addition, we expected those randomized to connection practices to show larger improvements in connection-related measures and those randomized to insight practice to show larger improvements in insight-related measures. We had several exploratory secondary hypotheses. We hypothesized that app usage would be positively associated with reduced distress. We hypothesized that improvements in connection- and insight-related measures would mediate effects on distress for those in the Connection and Insight arms, respectively. We hypothesized that those lower in mindfulness at baseline would show larger improvements in the active conditions and that those lower in connection- and insight-related measures would show larger improvements in the Connection and Insight arms, respectively. These hypotheses were preregistered at the Open Science Framework [68].

## Methods

### Procedure

We conducted an 8-week, fully remote RCT comparing 2 active smartphone-based meditation interventions with a waitlist control. Participants were recruited through emails sent to faculty, staff, and students at the University of Wisconsin-Madison and through a database of individuals who had previously expressed interest in research at the Center for Healthy Minds. All screening procedures and data collection were web-based and carried out using REDCap [69]. Participants completed a screening protocol to determine eligibility and received their group assignment via an automated email following the completion of baseline questionnaires. Randomization was achieved by automatically allocating participants to groups based on sequentially assigned participant identification numbers (ie, 1:1:1 randomization ratio). Participants were contacted by email to complete questionnaires 4 and 8 weeks postbaseline.

Progress through the material in the Healthy Minds Program (HMP) app was self-guided. There was minimal contact with the study staff. Participants were provided with a study email address to contact for technical support or study-related questions. All procedures were approved by the institutional review board. The study was registered at ClinicalTrials.gov (NCT04139005).

### Participants

Eligible participants were aged ≥18 years, had access to a smartphone or other device capable of running the intervention app (Android or iOS), and did not have extensive previous meditation experience defined as meditation retreat experience, meditation practice weekly for >1 year or daily practice within the previous 6 months; or previous training under the instruction of a meditation teacher, other than an introductory course. Participants received US $25 for completing the assessments.

### Intervention

Participants assigned to one of the 2 active intervention arms were instructed to download the HMP app through the Google Play or Apple App Store. The full HMP app includes 4 modules with practices designed to cultivate categories of mental and emotional skills linked to both hedonic and eudaimonic well-being [70,71]. These include the cultivation of mindful attention (Awareness), positive relationships with self and others (Connection), insight into the nature of self and internal experience (Insight), and purpose, values, and meaning in life (Purpose). In this study, the 2 active interventions included 4 weeks of Awareness training, followed by 4 weeks of either Connection or Insight training. This design was predicated on the view that training in the stabilization of attention is foundational to skills trained by Connection and Insight [72]. Each module included brief, podcast-style didactic material along with guided meditation practices. Didactic content included discussion of the scientific bases of the practices. Participants were encouraged to follow a prespecified sequence while going through the material. Participants could select the length of the guided practices (5-30 min) and a variety of practices were available in each module. For example, the Awareness module included practices focused on awareness of breathing and mindfulness of sound. The Connection module included gratitude and kindness practices. The Insight module included practices involving noticing the changing nature of the phenomenon (ie, impermanence) and examining how thoughts and emotions influence perception. Participants in the waitlist condition received access to the full HMP app (ie, all 4 modules) at the conclusion of the study.

### Measures

A demographic questionnaire was completed at baseline. App usage was measured objectively using the HMP app. Additional information about the psychometric properties and theoretical relevance of the included measures is provided in Multimedia Appendix 1 [73-101].

#### Psychological Distress

A psychological distress composite score was created from measures of depression, anxiety, and stress. We computed the mean across scaled (z-transformed) scores for each measure. The 8-item Patient-Reported Outcome Measures Information System Depression and Anxiety Scales [73] assessed depression and anxiety. Items are rated on a 5-point scale (1=never; 5=always), with higher scores indicating greater severity in the past 7 days. T scores ≥55 suggest mild or greater severity [102,103]. Internal consistency was high (=.93 to .94).

The 14-item Perceived Stress Scale [74] assessed psychological stress. Items are rated on a 5-point scale (0=never; 4=very often), with higher scores indicating greater stress in the past month. Internal consistency was high (=.89).

#### Measures Related to the Connection Module

The 20-item Social Connectedness Scale-Revised [75] assessed interpersonal connections. Items are rated on a 6-point scale (1=strongly disagree; 6=strongly agree), with higher scores indicating higher social connectedness. Internal consistency was high (=.95).

The 28-item Interpersonal Reactivity Index (IRI) [76] assessed empathy. Items are rated based on how well they describe the respondent on a 5-point scale (0=not well; 4=very well), with higher scores indicating greater empathy. Internal consistency was high for the total score (=.83).

The 21-item Compassionate Love Scale [77] assesses feelings of compassion. Items are rated on a 7-point scale (1=not at all true of me; 7=very true of me). Higher scores indicate greater feelings of compassion. Internal consistency was high (=.95).

#### Measures Related to the Insight Module

The 20-item Self-Reflection and Insight Scale (SRIS) [78] assessed participants’ tendency toward self-reflection (eg, “I frequently examine my feelings”) and self-understanding or insight (eg, “I usually know why I feel the way I do”). Items are rated on a 6-point scale (1=strongly disagree; 6=strongly agree) and yield subscales for self-reflection and insight, with higher scores indicating greater self-reflection or insight. Internal consistency was high (=.88 to .92).

The 15-item Perseverative Thinking Questionnaire (PTQ) [79] assessed rumination. For simplicity, we used the term “rumination” to refer to repetitive negative thinking as captured by the PTQ, although it captures both rumination and worry. Items are rated on a 5-point scale (0=never; 4=almost always), with higher scores indicating greater rumination. Internal consistency was high (=.96).

The 10-item Drexel Defusion Scale [80] assessed the ability to achieve psychological distance from internal experiences (ie, defusion). Items are rated on a 6-point scale (0=not at all; 5=very much), with higher scores indicating greater defusion. Internal consistency was high (=.89).

#### Mindfulness

The 39-item Five Facet Mindfulness Questionnaire [81] assessed mindfulness. Items are rated on a 5-point scale (1=never or very rarely true; 5=very often or always true), with higher scores indicating greater mindfulness. Internal consistency was high for the total score (=.94).

### Data Analysis

Results from all preregistered primary and secondary measures are reported. For deviations made from the preregistered data analytic plan, see Multimedia Appendix 1.

Data were analyzed using intention-to-treat principles (ie, participants were not excluded based on engagement) [104]. Primary analyses used multilevel models (MLMs [82] with restricted information maximum likelihood estimation in the *lme4* package [105] in R [106]. MLMs with a maximum likelihood estimator are generally robust to data that are missing at random (MAR) [83]. For each outcome, an MLM was specified in which a linear change (coded as 0, 1, 2, for pre-, mid-, and posttest, respectively) in outcome was assumed over time, with participant-level random intercepts. Intervention effects were evaluated by the interaction between linear growth and group status, with contrasts comparing the 2 active conditions (ie, Connection, Insight) as well as the combined active conditions relative to waitlist control (see Multimedia Appendix 1 for the model). A subsequent sensitivity analysis restricted the sample to participants above the clinical cut-off for depression or anxiety at baseline (T≥55) [103]. Sensitivity analyses were also conducted with outliers (ie, 3 SD from the mean) and each participant sequentially removed [84].

Additional analyses assessed the potential impact of attrition, which is common in fully remote RCTs [107]. In this study, it is plausible that missingness was related to the unobserved value itself (ie, missing not at random [MNAR]). For example, individuals who failed to benefit from the HMP app may have been less likely to complete the study and would have shown worse outcomes had they been observed. Therefore, we relaxed our MAR assumptions to evaluate the degree to which intervention effects would be maintained under MNAR assumptions. We examined intervention effects in the presence of different assumed outcomes for dropout-missing observations, focusing on residualized change scores (from baseline to posttest) to simplify the study of missingness implications. We coded outcomes for dropout missingness at different levels, ranging from no difference in outcomes (relative to those that remained in the study) to all dropout-missing values being the worst possible outcome of those in the study. As, operationally, it becomes easier to study this range of conditions using outcome ranks as opposed to retaining the metrics of the studied measures, we applied a nonparametric Wilcoxon rank sum test to compare the active conditions against the waitlist control under different missingness assumptions.

To test our exploratory mediation hypotheses, we used the *mediation* package in R [108]. In these models, active group status (Connection or Insight) served as the independent variable; pre-post changes in mindfulness or connection- or insight-related measures served as the mediators; and posttest distress (controlling for pretest) served as the dependent variable. Pre-post changes were examined as mediators as unique Connection and Insight content was provided after the midtreatment assessments. We used MLMs to examine the effect of app usage, testing the time×usage interaction with usage operationalized as the median split of days of use. As noted in Multimedia Appendix 1, a median split was used because of deviations from normality in usage metrics. To assess baseline characteristics as moderators of change in distress, we tested 3-way interactions between time, group, and baseline characteristics within MLMs. False discovery rate (FDR) adjustment [85] was applied to all analyses to control for inflation of a type I error.

### Sample Size and Power

We planned to recruit 300 participants (100 per group), which would allow the detection of small-to-moderate differences between any 2 groups (*d*=0.40) and between the active and waitlist control conditions (*d*=0.34) at 80% power and *P*=.05. Power was estimated using the *pwr.t.test* and *pwr.t2n.test* functions in the *pwr* package in R [109].

## Results

### Recruitment and Participant Characteristics

**Figure 1:**
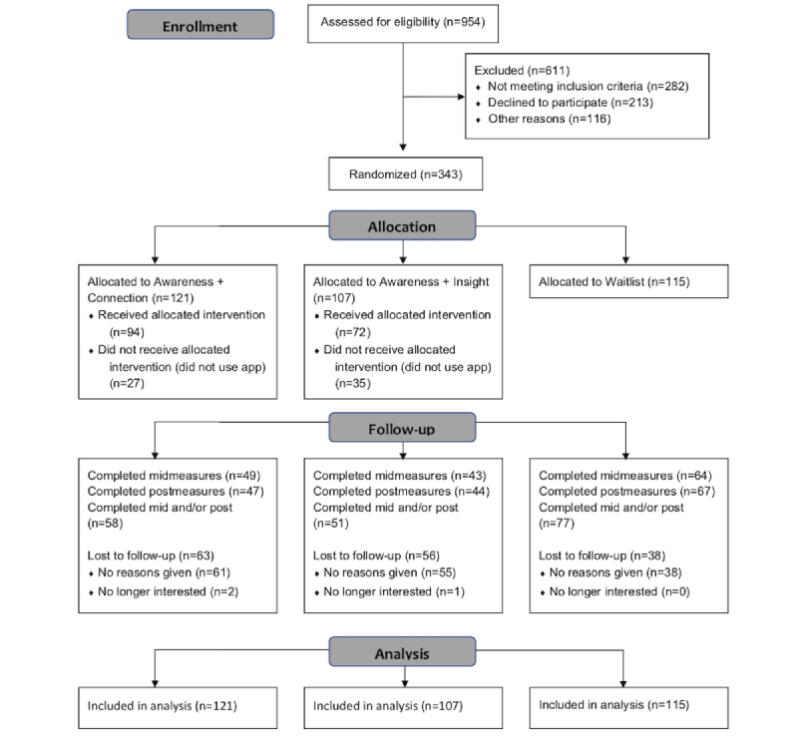
CONSORT (Consolidated Standards of Reporting Trials) diagram. Mid=week 4 assessment; Post=week 8 assessment.

A total of 954 potential participants were assessed for eligibility, of which 343 met the inclusion criteria and were randomized to Connection (n=121), Insight (n=107), or waitlist (n=115; Figure 1). Demographics are reported in Table 1. The sample was predominantly White (280/343, 81.6%), female (290/343, 84.5%), and with graduate-level education (190/343, 55.4%). Income was more variable (89/343, 25.9% earned US $50,000 or less). The mean age was 41.74 (SD 12.52) years.

**Table 1:** Sample demographics.

Variable			Overall (n=343)		Connection (n=121)		Insight (n=107)		Waitlist (n=115)		*P* value^a^
**Race and ethnicity, n (%)**										.91	
	White	280 (81.6)		99 (81.8)		86 (80.4)		95 (82.6)			
	Black	6 (1.7)		3 (2.5)		1 (0.9)		2 (1.7)			
	Latinx	4 (1.2)		0 (0.0)		4 (3.7)		0 (0.0)			
	Asian	18 (5.2)		7 (5.8)		6 (5.6)		5 (4.3)			
	Multiracial	33 (9.6)		12 (9.9)		9 (8.4)		12 (10.4)			
	Not reported	2 (0.6)		0 (0.0)		1 (0.9)		1 (0.9)			
**Gender, n (%)**										.93	
	Female	290 (84.5)		101 (83.5)		89 (83.2)		98 (85.2)			
	Male	51 (14.9)		20 (16.5)		15 (14.0)		16 (13.9)			
	Nonbinary	2 (0.6)		0 (0.0)		1 (0.9)		1 (0.9)			
	Not reported	2 (0.6)		0 (0.0)		2 (1.9)		0 (0.0)			
**Income (US $), n (%)**										.18	
	≤50,000	89 (25.9)		33 (27.3)		26 (24.3)		30 (26.1)			
	50,000-100,000	120 (35.0)		35 (28.9)		37 (34.6)		48 (41.7)			
	100,000-150,000	76 (22.2)		32 (26.4)		22 (20.6)		22 (19.1)			
	>150,000	57 (16.6)		21 (17.4)		21 (19.6)		15 (13.0)			
	Not reported	1 (0.3)		0 (0.0)		1 (0.9)		0 (0.0)			
**Education, n (%)**										.45	
	Some high school	1 (0.3)		0 (0.0)		1 (0.9)		0 (0.0)			
	High school graduate	6 (1.7)		3 (2.5)		0 (0.0)		3 (2.6)			
	Some college	32 (9.3)		9 (7.4)		11 (10.3)		12 (10.4)			
	College graduate	114 (33.2)		37 (30.6)		36 (33.6)		41 (35.7)			
	Graduate school	190 (55.4)		72 (59.5)		59 (55.1)		59 (51.3)			
Age (years), mean (SD)			41.74 (12.52)		42.31 (12.8)		43.21 (12.39)		39.78 (12.2)		.10
Elevated symptoms^b^, n (%)			252 (73.5)		86 (71.1)		81 (75.7)		85 (73.9)		.73

^a^*P* values based on a one-way analysis of variance with group (Connection, Insight, or waitlist) predicting demographics (White, female, high income [≥US $100,000], and graduate school).

^b^Elevated symptoms: Patient-Reported Outcomes Measurement Information System (PROMIS) Depression or PROMIS Anxiety in the mild or higher range (T≥55).

### Utilization

Of those randomized to one of the 2 active conditions, 77.7% (94/121) of Connection participants and 67.3% (72/107) of Insight participants downloaded and used the HMP app at least once. By assigning values of zero to those who did not use the app, we found that average utilization was 10.52 days (SD 13.31; median 4), with 18.09 activities within the app (SD 23.30; median 7), 9.45 meditation practices (SD 13.34; median 3), and 102.16 total min of meditation practice (SD 187.74; median 26). All usage metrics were highly zero inflated (Multimedia Appendix 1). Days of use had the lowest skewness (1.34) and kurtosis (0.91), so a median split of days of use was used in the analyses. The median survival time (ie, time before last use) was 12 days. Group status (Connection vs Insight) was not associated with usage (*P*=.15), and survival time did not differ between groups (Multimedia Appendix 1; *P*=.24). As Connection or Insight content was provided at week 5 of the program, 32.2% (39/121) of Connection and 23.4% (25/107) of Insight participants engaged with the unique content. This proportion did not differ between groups (OR 0.64, 95% CI 0.35-1.15; *P*=.14).

### Attrition Analysis

We examined baseline demographic and outcome variables as predictors of attrition. We constructed logistic regression models predicting the presence of any follow-up data (ie, midtreatment or posttest). Participants were invited to complete the posttest measures even if they had not completed the midtreatment measures. The average completion of at least one follow-up assessment (mid- or posttreatment) was 54.2% (186/343). Waitlist participants were more likely to complete follow-up assessments (77/115, 67.0% vs 109/228, 47.8%; waitlist n=77; Connection and Insight combined n=109; OR 2.21, 95% CI 1.39-3.56; *P*<.001). Completion of follow-up assessments did not differ between the Connection and Insight groups (OR 0.99, 95% CI 0.59-1.67; *P*=.97). However, participants who used the app at least once were more likely to complete the follow-up assessments (OR 3.66, 95% CI 1.95-7.16]; *P*<.001). Completion of follow-up was not associated with demographics (White, female, high income [≥US $100,000], and graduate education) or outcome measures at baseline (*P*s≥.15), with one exception. Participants with higher empathy scores (IRI) at baseline were more likely to complete follow-up assessments (OR 1.02, 95% CI 1.00-1.04; *P*=.04).

### Primary Analyses

**Figure 2:**
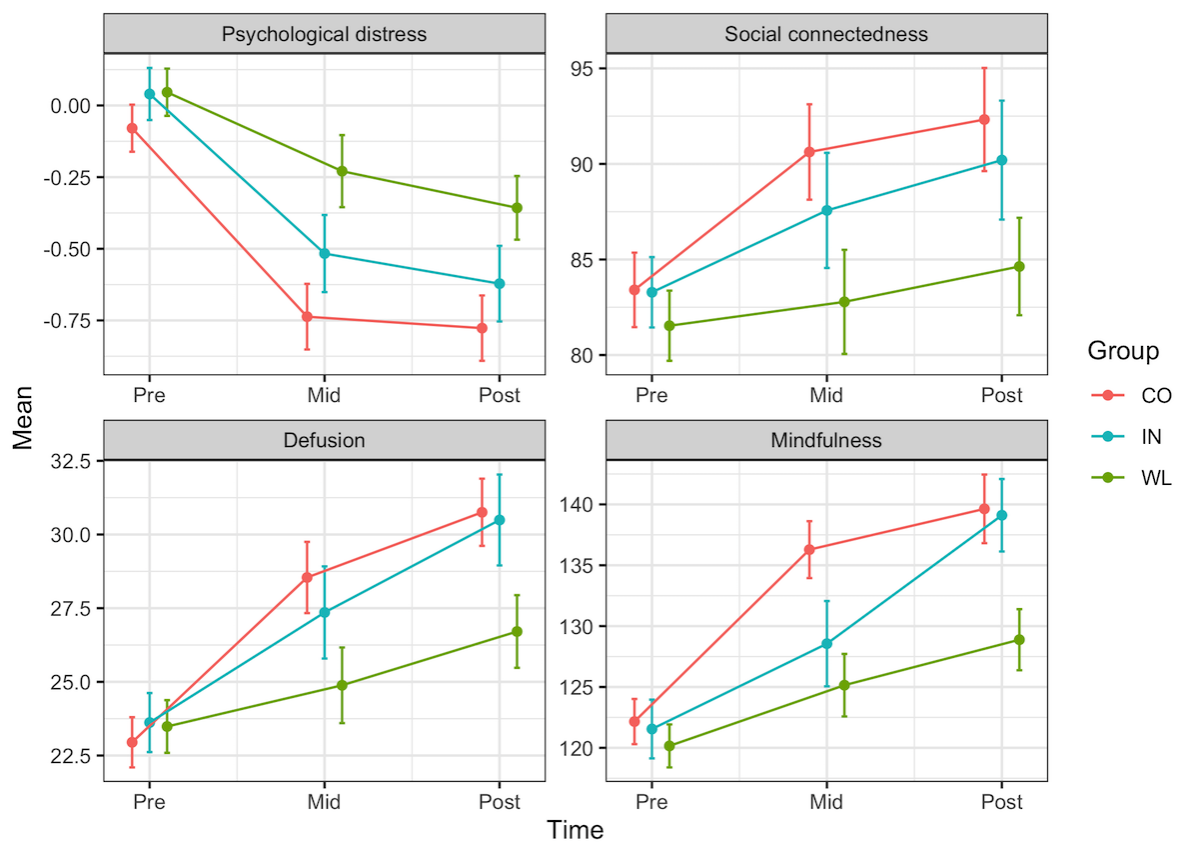
Longitudinal changes in psychological distress, social connectedness, defusion, and mindfulness by group. The figure displays observed means and SEs (error bars=1 SE) based on all available data (n=343). CO: Awareness+Connection; IN: Awareness+Insight; WL: waitlist.

Correlations between outcomes are reported in Multimedia Appendix 1. The 3 groups did not differ in any demographic or outcome measures at baseline (*P*s≥.10; Tables 1 and 2). Within- and between-group effect sizes (Cohen *d*) and *P* values from MLMs are reported in Table 3. The 2 active conditions did not differ from one another in terms of change over time for distress or any secondary outcomes (time×group, *P*s≥.29). Therefore, all subsequent analyses combined the 2 active groups. When compared with the waitlist control, the active conditions showed greater decreases in distress (*d*=0.28) and rumination (*d*=0.18) and greater increases in social connectedness, self-reflection, insight, defusion, and mindfulness (*d*=0.13 to 0.41; FDR-adjusted *P*s≤.02; Figure 2). The active conditions did not differ from the waitlist on changes in empathy (*d*=0.02) or compassion (*d*=0.12). Significance tests for time×group interactions did not change when restricting to those with elevated symptoms at baseline (Table 2), when excluding outliers (with the exception of Self-Reflection, *P*=.05; Multimedia Appendix 1), nor when each case was excluded sequentially.

A larger proportion of participants in the active conditions showed a minimally important decrease in distress (*d*≤–0.30) [86] relative to the waitlist condition (70% vs 49%; Connection and Insight combined n=64/91; waitlist n=33/67; OR 2.44 [95% CI 1.27-4.75]; *P*=.008). A smaller proportion in the active condition showed a minimally important increase in distress (ie, deterioration, *d*≥0.30) relative to the waitlist condition (3% vs 16%; Connection and Insight combined n=3/91; waitlist n=11/67; OR 0.17 [0.04, 0.58]; *P*=.009).

**Table 2:** Descriptive statistics for repeated measures by group and timepoint.

Group and outcome			Pretest			Midtreatment				Posttest				*P* value^a^	
			n		Mean (SD)		n		Mean (SD)		n		Mean (SD)		
**CO^b^**															
	Psychological distress^c^	121		0.08 (0.90)		49		0.74 (0.80)		47		0.78 (0.78)		.49	
	Social connection^d^	121		83.41 (21.43)		48		90.62 (17.29)		46		92.32 (18.30)		.73	
	Empathy^e^	121		67.67 (12.33)		48		67.42 (12.55)		47		66.01 (12.90)		.41	
	Compassion^f^	121		99.18 (22.19)		48		102.52 (21.71)		46		101.68 (21.64)		.57	
	Self-reflection subscale^g^	121		56.31 (11.13)		48		58.45 (10.29)		46		57.49 (11.10)		.97	
	Insight subscale^g^	121		34.12 (7.46)		48		37.06 (6.17)		46		37.54 (6.20)		.46	
	Rumination^h^	121		31.12 (12.65)		46		25.31 (9.63)		46		25.36 (10.71)		.90	
	Defusion^i^	121		22.95 (9.37)		48		28.54 (8.37)		47		30.75 (7.81)		.86	
	Mindfulness^j^	121		122.16 (20.41)		49		136.28 (16.37)		47		139.63 (19.33)		.77	
**IN^k^**															
	Psychological distress	107		0.04 (0.94)		43		0.52 (0.88)		44		0.62 (0.88)		N/A^l^	
	Social connection	107		83.28 (19.04)		41		87.57 (19.28)		44		90.20 (20.64)		N/A	
	Empathy	107		69.77 (12.45)		41		70.24 (11.76)		44		69.49 (11.05)		N/A	
	Compassion	107		101.57 (21.44)		41		101.07 (24.52)		44		108.88 (23.29)		N/A	
	Self-reflection subscale	107		55.99 (10.40)		41		55.90 (9.00)		44		57.92 (9.45)		N/A	
	Insight subscale	107		32.84 (8.42)		41		33.67 (8.28)		44		36.14 (8.44)		N/A	
	Rumination	107		30.49 (11.71)		41		27.21 (10.79)		44		23.97 (10.68)		N/A	
	Defusion	107		23.62 (10.36)		41		27.36 (10.01)		44		30.49 (10.23)		N/A	
	Mindfulness	107		121.55 (24.96)		43		128.56 (22.96)		44		139.11 (19.75)		N/A	
**WL^m^**															
	Psychological distress	115		0.05 (0.88)		64		0.23 (1.01)		67		0.36 (0.91)		N/A	
	Social connection	115		81.53 (19.68)		60		82.78 (21.10)		63		84.63 (20.24)		N/A	
	Empathy	115		69.31 (12.85)		60		71.09 (13.49)		63		67.96 (13.26)		N/A	
	Compassion	115		98.62 (22.39)		58		99.53 (22.64)		63		100.85 (22.42)		N/A	
	Self-reflection subscale	115		56.34 (10.78)		57		56.87 (11.14)		63		56.23 (11.26)		N/A	
	Insight subscale	115		33.22 (8.02)		57		35.40 (8.29)		63		35.55 (7.83)		N/A	
	Rumination	115		31.14 (11.56)		56		29.51 (13.04)		62		27.41 (11.92)		N/A	
	Defusion	115		23.49 (9.58)		60		24.88 (9.95)		64		26.71 (9.86)		N/A	
	Mindfulness	115		120.16 (18.93)		62		125.15 (20.21)		65		128.88 (20.23)		N/A	

^a^*P* value from a one-way analysis of variance predicting baseline values for outcome measures by group status.

^b^CO: Awareness+Connection.

^c^Composite of Patient-Reported Outcomes Measurement Information System (PROMIS) Depression, PROMIS Anxiety, and Perceived Stress Scale.

^d^Social Connectedness Scale.

^e^Interpersonal Reactivity Index.

^f^Compassionate Love Scale.

^g^Subscales of the Self-Reflection and Insight Scale.

^h^Perseverative Thinking Questionnaire.

^i^Drexel Defusion Scale.

^j^Total score of Five Facet Mindfulness Questionnaire.

^k^IN: Awareness+Insight.

^l^N/A: not applicable.

^m^WL: waitlist.

**Table 3:** Results of multilevel models assessing differential change over time.

Outcome	CO^a^ versus IN^b^					Active versus WL^c^							
	*d*_CO_^d^	*d*_IN_	*d*_diff_	*P* value^e^	*P*_FDR_^f^		*d*_Active_^g^	*d*_WL_	*d*_diff_	*P* value	*P*_FDR_	Elev *P*^h^	Elev *P*_FDR_
Psychological distress^i^	–0.77	–0.70	–0.07	.86	.97		–0.74	–0.46	–0.28	<.001	<.001	<.001	<.001
Social connection^j^	0.42	0.36	0.06	.54	.82		0.39	0.16	0.23	.003	.007	.01	.02
Empathy^k^	–0.14	–0.02	–0.12	.37	.82		–0.08	–0.10	0.02	.63	.63	.48	.48
Compassion^l^	0.11	0.34	–0.23	.29	.82		0.22	0.10	0.12	.14	.16	.18	.20
Self-reflection subscale^m^	0.11	0.18	–0.07	.51	.82		0.14	–0.01	0.15	.007	.01	.02	.03
Insight subscale^m^	0.46	0.39	0.07	.98	.98		0.42	0.29	0.13	.02	.02	.001	.002
Rumination^n^	–0.45	–0.56	0.11	.32	.82		–0.5	–0.32	–0.18	.01	.02	.007	.01
Defusion^o^	0.83	0.66	0.17	.78	.97		0.75	0.34	0.41	<.001	<.001	<.001	<.001
Mindfulness^p^	0.86	0.70	0.16	.55	.82		0.77	0.46	0.31	<.001	<.001	<.001	<.001

^a^CO: Awareness+Connection.

^b^IN: Awareness+Insight.

^c^WL: waitlist.

^d^Cohen *d* calculated as pre-post for within-group effects and the difference between within-group effects (Connection−Insight, active−waitlist) for *d*_diff_. For within-group, subscripted CO (ie, *d*_CO_), IN, Active, and WL refer to subgroups noted.

^e^*P* value from time×group interaction from multilevel models.

^f^FDR: false discovery rate adjusted *P* values.

^g^Combined Awareness+Connection and Awareness+Insight.

^h^Active versus waitlist time×group interaction restricted to sample with elevated depression or anxiety at baseline (T≥55).

^i^Composite of Patient-Reported Outcomes Measurement Information System (PROMIS) Depression, PROMIS Anxiety, and Perceived Stress Scale.

^j^Social connection: Social Connectedness Scale.

^k^Interpersonal Reactivity Index.

^l^Compassionate Love Scale.

^m^Subscales of the Self-Reflection and Insight Scale.

^n^Perseverative Thinking Questionnaire.

^o^Drexel Defusion Scale.

^p^Total score of Five Facet Mindfulness Questionnaire.

### Robustness Check: Sensitivity Analyses

Although maximum likelihood is robust to data MAR [83], subsequent analyses evaluated treatment effects based on varying assumptions under MNAR conditions. Using the completer sample, a Wilcoxon rank sum test on the residualized gain score mirrored the MLM results, with larger improvements in the active conditions relative to the waitlist on several outcomes (FDR-adjusted *P*s≤.047; Multimedia Appendix 1). In the worst-case scenario model in which missing reflects the worst possible outcome across both active and waitlist groups, the groups did not differ, although the direction of the mean rank favored the waitlist group for all outcomes. Thus, we examined the results in between these extreme conditions to understand where significance goes away and where the direction of intervention effect reverses. When we assumed that missing values are on average 0.25 SD above the mean (implying worse than average outcomes for the missing observations), the results continued to favor the active conditions for changes in distress, social connectedness, defusion, and mindfulness (FDR-adjusted *P*s≤.04; Multimedia Appendix 1). When we assumed that missing values were on average 0.50 SD above the mean, the differences between groups were not statistically significant for any outcome. The difference remained nonsignificant when we assumed that missing values are on average 0.75 SD above the mean. Thus, it appears that our results are robust to MNAR up to a point, specifically that missing outcomes are no more than 0.25 SD above the mean on average, under the assumption that missingness implies comparable outcomes for both the active and waitlist groups.

### Secondary Analyses

**Figure 3:**
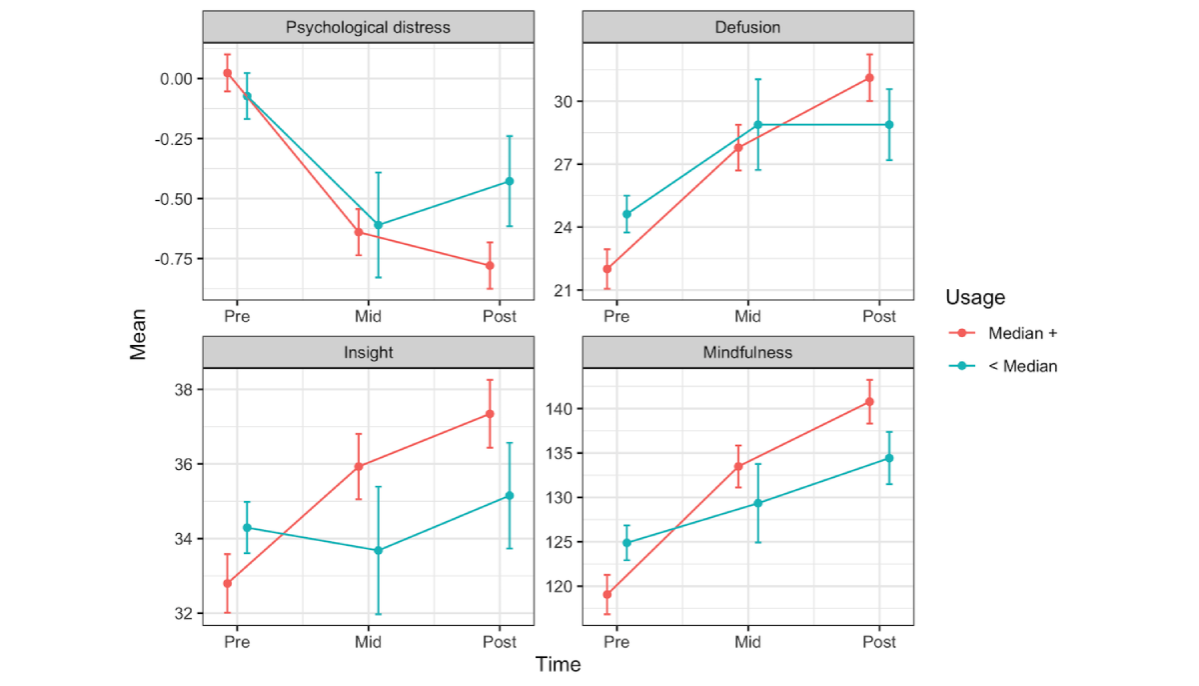
Healthy Minds Program app usage predicting longitudinal changes in psychological distress, defusion, insight, and mindfulness in active conditions (FDR-corrected Ps≤.04). Usage=median split of days of use (n=228). HMP: Healthy Minds Program; WL: waitlist.

The results of the usage analyses are reported in Table 4. HMP use above the median number of days was associated with larger improvements in distress, insight, defusion, and mindfulness (FDR-adjusted *P*s≤.03; Figure 3).

The results of the mediation analyses are reported in Table 5. Changes in 5 candidate mechanisms showed a significant average causal mediation effect (FDR-adjusted *P*s≤.04) in the expected direction (ie, improvements in social connection, insight, rumination, defusion, and mindfulness mediated improvements in distress). Changes in mindfulness were associated with the largest proportion mediated (0.45).

**Table 4:** Results of multilevel models predicting changes in outcomes from Healthy Minds Program app usage (n=228).

Outcome	Time×usage *B*^a^	*t* test (*df*)^b^	*P* value^c^	*P*_FDR_^d^
Psychological distress^e^	–0.17	–2.46 (207)	.02	.03
Social connection^f^	2.42	1.61 (202)	.11	.17
Empathy^g^	–0.47	–0.58 (200)	.56	.56
Compassion^h^	–1.16	–0.76 (201)	.45	.50
Self-reflection subscale^i^	0.80	0.87 (218)	.38	.49
Insight subscale^i^	1.55	2.46 (211)	.02	.03
Rumination^j^	–1.85	–2.13 (197)	.03	.06
Defusion^k^	2.40	2.64 (229)	.009	.03
Mindfulness^l^	5.17	2.93 (212)	.004	.03

^a^Multilevel model regression coefficient. Usage: days of use split into high (median or above) and low (below median) groups.

^b^*t* statistic for time×usage interaction with associated degrees of freedom (*df*).

^c^*P* value for time×usage interaction.

^d^FDR: false discovery rate adjusted *P* values.

^e^Composite of Patient-Reported Outcomes Measurement Information System (PROMIS) Depression, PROMIS Anxiety, and Perceived Stress Scale.

^f^Social Connectedness Scale.

^g^Interpersonal Reactivity Index.

^h^Compassionate Love Scale.

^i^Subscales of the Self-Reflection and Insight Scale.

^j^Perseverative Thinking Questionnaire.

^k^Drexel Defusion Scale.

^l^Total score of Five Facet Mindfulness Questionnaire.

**Table 5:** Results of mediation analyses predicting changes in psychological distress.

Outcome	ACME^a^	ADE^b^	Prop mediated^c^	*P* value^d^	*P*_FDR_^e^
Social connection^f^	–0.10	–0.32	0.24	.002	.005
Empathy^g^	0.02	–0.44	–0.03	.40	.47
Compassion^h^	0.01	–0.43	–0.01	.59	.59
Self-reflection subscale^i^	0.02	–0.44	–0.04	.41	.47
Insight subscale^i^	–0.06	–0.37	0.13	.03	.04
Rumination^j^	–0.10	–0.32	0.23	.02	.04
Defusion^k^	–0.10	–0.32	0.23	.002	.005
Mindfulness^l^	–0.18	–0.22	0.45	<.001	<.001

^a^ACME: average causal mediation effect (ie, indirect effect).

^b^ADE: average direct effect (ie, from active to posttreatment distress controlling for pretreatment distress, when active=1 and waitlist=0).

^c^Proportion mediated computed as indirect effect (ie, ACME) divided by total effect [108].

^d^*P* value based on quasi-Bayesian CIs.

^e^FDR: false discovery rate adjusted *P* values. Models examining pre-post change in constructs related to Awareness, Connection, and Insight modules as mediators of pre-post change in (composite of PROMIS Depression, PROMIS Anxiety, and Perceived Stress Scale). Proportion mediated can be negative in instances where direct effect and indirect effect have opposite signs.

^f^Social connection: Social Connectedness Scale.

^g^Interpersonal Reactivity Index.

^h^Compassionate Love Scale.

^i^Subscales of the Self-Reflection and Insight Scale.

^j^Perseverative Thinking Questionnaire.

^k^Drexel Defusion Scale.

^l^Total score of Five Facet Mindfulness Questionnaire.

The results of the baseline moderation analyses are reported in Table 6. A total of 3 baseline variables showed significant time×group×baseline interactions after FDR adjustment. Psychological vulnerability, as indicated by 2 outcomes (higher rumination and lower defusion) at baseline, was associated with significant improvements in distress in the HMP conditions relative to the waitlist condition. Baseline empathy showed the opposite pattern, with those higher at baseline showing significant improvements in distress in HMP relative to the waitlist condition (Multimedia Appendix 1).

**Table 6:** Baseline outcomes as moderators of longitudinal changes in psychological distress.

Outcome	Time×group *B*^a^	Time×group×baseline *B*	*t* test (df)^b^	*P* value^c^	*P*_FDR_^d^
Social connection^e^	–0.64	0.01	2.16 (363)	.03	.06
Empathy^f^	0.67	–0.01	–3.23 (343)	.001	.008
Compassion^g^	0.07	0.00	–1.20 (343)	.23	.29
Self-reflection subscale^h^	–0.59	0.01	1.55 (339)	.12	.19
Insight subscale^h^	–0.44	0.01	1.14 (359)	.25	.29
Rumination^i^	0.21	–0.01	–3.15 (390)	.002	.008
Defusion^j^	–0.54	0.01	2.86 (361)	.004	.01
Mindfulness^k^	–0.44	0.00	0.82 (372)	.41	.41

^a^Multilevel model regression coefficient.

^b^*t* test: *t* statistic for time×group×baseline (with group coded as active=1, waitlist=0) with associated degrees of freedom (df).

^c^*P* value for time×group×baseline.

^d^FDR: false discovery rate adjusted *P* values.

^e^Social Connectedness Scale.

^f^Interpersonal Reactivity Index.

^g^Compassionate Love Scale.

^h^Subscales of the Self-Reflection and Insight Scale.

^i^Perseverative Thinking Questionnaire.

^j^Drexel Defusion Scale.

^k^Total score of Five Facet Mindfulness Questionnaire.

## Discussion

### Principal Findings

This study sought to expand the scientific understanding of the impact of smartphone-delivered meditation training beyond mindfulness. To do so, we evaluated the effects of mindfulness training (Awareness) paired with practices designed to cultivate kindness toward oneself and others (Connection) or insight into the nature of self and internal experience (Insight). We assessed the effects on psychological distress and constructs theoretically linked to connection- and insight-based training [54].

Contrary to our expectations, there was no indication that training in connection produced differential effects relative to insight-related practices. There are several potential reasons for this. One likely explanation is that the actual content completed by each group was largely overlapping. Both groups began with foundational mindfulness training. Given the modest engagement (a perennial concern in mHealth interventions) [110,111], most participants did not engage with the unique Connection or Insight modules. It is also possible that meditation training produces similar effects for novices, regardless of the specific type of training. Novices may spend much of their initial meditation practice simply regathering a wandering attention, regardless of the actual practice instructions. Indeed, studies showing distinct neural signatures associated with various forms of meditation practice have primarily been conducted with long-term practitioners with thousands of hours of experience [55]. A third possibility is that various forms of meditation training contain common ingredients (eg, acceptance, curiosity) that may, especially early in training, be more potent than style-specific ingredients.

Despite the absence of differential effects, the results suggest that meditation delivered via smartphones produced small reductions in psychological distress (*d*=–0.28) and improvements in several candidate mechanisms relative to a waitlist control (*d*=–0.18 to 0.41). These results are generally consistent with meta-analyses of the broader mHealth and mHealth MBI literature, which has shown small benefits of self-guided smartphone apps on depression and anxiety symptoms (*g*=0.21 to 0.23) and measures of mindfulness and acceptance (*g*=0.27) [30]. These effects are considerably smaller than those produced by in-person MBIs (eg, *d*=0.55 vs waitlist) [9]. It is likely that mHealth MBIs may be less potent than in-person interventions, indicating trade-offs between scalability, cost, and potency. On the basis of those completing posttreatment measures, HMP appears safe in that the rates of clinically significant increases in distress were rare (3%) and were less common than the rates in the control condition (16%). This finding is consistent with a recent large-scale evaluation of the deterioration in MBSR [87].

One important caveat for interpreting our findings is high attrition, particularly within the active conditions. Both high attrition and differential attrition are common in mHealth research [107,112]. Our overall attrition rate was almost identical to that typically found in RCTs testing smartphone interventions without telephone or in-person enrollment (45.8% in this study and 43.4% in the meta-analysis) [107]. In addition to employing maximum likelihood estimation in all MLMs (which is robust to MAR) [83], we conducted a series of sensitivity analyses to assess the effects of various MNAR assumptions. Most effects were robust to noncompleters having outcomes slightly worse than completers (SD 0.25). However, the effects did not persist with larger deviations (SD ≥0.50). It is impossible to directly test which of these scenarios is most likely (as is the case for other MNAR approaches) [113]. Future studies should include items specifically to predict missingness (eg, “how likely are you to drop out of this study”) [114]. Responses can then be included as auxiliary variables to improve the performance of MAR methods (effectively converting MNAR to MAR) [114].

In light of the degree of attrition, secondary analyses should be interpreted as exploratory. However, these models provide tentative possibilities to be examined further. We found evidence that higher usage (median or above days of use) was associated with larger improvements in distress and several other outcomes. This mirrors the dosage-outcome associations seen in the in-person MBI literature [115]. Mediation analyses suggest candidate mechanisms theoretically linked to each HMP module that may indirectly contribute to decreased distress (ie, mindfulness, social connectedness, defusion, rumination). This also mirrors reviews of the in-person MBI literature that have found changes in mindfulness and rumination mediate effects [116]. The possibility that social connectedness also plays a role should be explored further, particularly as it has been associated with numerous psychological and physical health outcomes [117,118]. Moderation analyses indicated larger improvements among those showing higher rumination or empathy and lower defusion at baseline. These are somewhat conflicting findings, with the rumination and defusion associations suggesting that HMP may be most effective for those with deficits at baseline, whereas the association with empathy suggesting a higher baseline level may be necessary to benefit most. Given that mHealth interventions could, in theory, be easily adapted to participant characteristics (eg, participants routed to receive a particular version based on baseline questionnaires), future experimental work can specifically examine who is likely to benefit from which kind of training (eg, randomizing to adapted vs nonadapted versions). The scalability of mHealth RCTs may allow recruitment of the sample sizes necessary for adequately powered tests of moderation [119].

### Limitations and Future Directions

As noted, high and differential attrition are limitations of this study. Although attrition is commonplace in mHealth research [110], future studies should include study design features that decrease attrition (eg, telephone enrollment) [107]. Relatedly, engagement with the HMP app was relatively modest. Similar to attrition, low engagement is a rule rather than an exception for mHealth [120]. Presumably low engagement diminished any potential effects of the HMP app, making estimates of efficacy more ambiguous. Future studies could evaluate engagement strategies by randomizing participants to receive approaches found to improve adherence to medical regimens (eg, modifying dosage recommendations, providing reminders) [121]. Microrandomized trials could assess the impact of a variety of small manipulations on short-term engagement outcomes [122].

The lack of a follow-up assessment is another limitation, making it unclear whether any benefits were sustained. Furthermore, the lack of active control conditions makes it impossible to rule out effects due to a digital placebo [123]. Similarly, the included self-report measures are vulnerable to social desirability bias, although this may be less of an issue within a fully remote RCT [124]. Sampling procedures and sample demographics raise questions regarding generalizability, especially to racial/ethnic minority populations and those with lower levels of education. Participants in the Center for Healthy Minds database may have been particularly amenable to the HMP app (although those with prior meditation experience would have been excluded).

An obvious future study would assign participants to receive only Connection or Insight module content. This could clarify the unique effects of these types of practices. As we observed effects on distress that persisted when restricted to those with elevated symptoms at baseline, it would be worthwhile replicating this study within a clinical sample. For this, it could be important or even necessary, for safety reasons, to include some amount of professional guidance [41], perhaps telephone or text-based support [125]. Other extensions of this study could include the use of non–self-report measures, both to rule out social desirability as well as to clarify underlying mechanistic processes. Numerous biological and behavioral mechanisms have been assessed for in-person MBIs (eg, changes in attention, body awareness, stress physiology) [126-128] and may be operant within mHealth MBIs. A wide variety of extensions could also be made to the HMP app itself. For example, intervention components could be provided in response to passively assessed mood states (eg, using data streams routinely gathered through phone sensors). This would require not only the validation of passive measures [129,130] but also studies that clarify the optimal pairing of intervention components to mood. Microrandomized trials may be an ideal design for this purpose. The amount of engagement necessary for clinical benefits (ie, recommended dosage) should be clarified in future studies (eg, by random assignment to dosage conditions). RCTs using active control conditions can help clarify the role that nonspecific factors may play in mHealth MBIs. On the basis of the robust association between working alliances and outcomes within in-person interventions [131], future studies should assess its digital corollary [132]. Finally, a critical future direction is investigating the efficacy of mHealth MBIs specifically among (and ideally tailored for) [133,134] racial/ethnic minorities. Racial/ethnic minority populations are at increased risk for racism-related negative psychological and physical health consequences [135] and have been historically underrepresented in research on mindfulness [136,137].

### Conclusions

Research into the mobile delivery of meditation training has expanded rapidly in the past five years. However, the vast majority of this work has focused exclusively on training mindfulness. We found preliminary evidence that 2 versions of a novel smartphone app that includes training in mindfulness, in addition to skills specifically targeted to improve social connection and intrapersonal insight, are associated with reduced psychological distress, increased social connectedness, and improvements in candidate mechanisms believed to underlie the beneficial effects of MBIs. Future studies, particularly those focusing on decreasing study dropout and increasing intervention engagement, are warranted.

## Appendix

Multimedia Appendix 1 Healthy Minds Program randomized controlled trial supplemental materials.

Multimedia Appendix 2 CONSORT-eHealth (V 1.6.1).

## Abbreviations

FDR: false discovery rate
HMP: Healthy Minds Program
IRI: Interpersonal Reactivity Index
MAR: missing at random
MBCT: mindfulness-based cognitive therapy
MBI: meditation-based intervention
MBSR: mindfulness-based stress reduction
mHealth: mobile health
MLM: multilevel model
MNAR: missing not at random
PTQ: Perseverative Thinking Questionnaire
RCT: randomized controlled trial
