# Supplementary material for: Testing the Efficacy of a Multicomponent, Self-Guided, Smartphone-Based Meditation App: Three-Armed Randomized Controlled Trial
Source: JMIR Ment Health. 2020 Nov 27;7(11):e23825. doi: 10.2196/23825 (PMC7732708; doi:10.2196/23825)
Supplement: Multimedia Appendix 1 [file mental_v7i11e23825_app1.docx]

**Multimedia Appendix 1**

Table 1. Additional psychometric information for the included self-report measures

**PROMIS Depression and PROMIS Anxiety**[1]

Both measures were developed using item response theory to maximize information provided while minimizing measure length. Sample items include “I felt worthless” (depression) and “I felt fearful” (anxiety). Both measures have shown strong convergent validity with legacy measures[2,3].

**Perceived Stress Scale (PSS)**[4]

A sample item is “how often have you been upset because of something that happened unexpectedly?” The measure is widely used and has shown desirable psychometric properties[5].

**Social Connectedness Scale-Revised (SCS-R)**[6]

A sample item is “I feel close to people.” The measure has been previously linked to well-being[7]. This measure has shown strong reliability and validity[8] and has previously shown responsiveness to connection-related practices[9].

**Interpersonal Reactivity Index (IRI)**[10]

The IRI has four subscales. Sample items from each subscale include “I try to look at everybody’s side of a disagreement before I make a decision” (perspective taking), “I am often quite touched by things I see happen” (empathic concern), “I really get involved with the feelings of characters in a novel” (fantasy), and “I tend to lose control during emergencies” (personal distress). Based on factor analyses suggesting a single overarching factor[11] and consistent with prior studies[12], we calculated a total score from all items.

**Compassionate Love Scale** **(CLS)**[13]

A sample item is “I spend a lot of time concerned about the well-being of humankind.” The CLS has shown discriminant validity relative to measures of empathy and predicts prosocial behavior when controlling for empathy[13].

**Self-Reflection and Insight Scale (SRIS)**[14]

The SRIS has two subscales. Sample items from each subscale include “I frequently examine my feelings” (self-reflection) “I usually know why I feel the way I do” (insight). Scores on the insight subscale have been found to partially mediate cross-sectional associations between mindfulness and well-being[15].

**Perseverative Thinking Questionnaire** **(PTQ)**[16]

A sample item is “my thoughts take up all my attention.” The measure has shown high internal consistency and test-retest reliability and is designed to detect transdiagnostic processes associated with clinical diagnoses of anxiety and depression[16]. Assessment of rumination was included as a measure of changes anticipated following Insight training because it includes practices that encourage thoughts, particularly negative thoughts about oneself, to be experienced as constructed mental events rather than actual depictions of reality, which can foster a more flexible and dynamic self-narrative and less rumination.

**Drexel Defusion Scale (DDS)**[17]

A sample item is “Imagine you bang your knee on a table leg. To what extent would you normally be able to defuse from physical pain?” In order to address concerns that measures of similar constructs are often misunderstood (e.g., mindfulness)[18], the DDS provides an extended definition of the term defusion. The DDS has high convergent validity with measures of acceptance and psychological distancing and incremental validity in predicting psychopathology and quality of life.

**Five Facet Mindfulness Questionnaire (FFMQ)**[19]

The FFMQ has five subscales. Sample items from each subscale include “When I’m walking, I deliberately notice the sensations of my body moving” (observe), “I’m good at finding words to describe my feelings” (describe), “When I do thinking, my mind wanders off and I’m easily distracted” (acting with awareness), “I perceive my feelings and emotions without having to react to them” (non-reactivity), and “I criticize myself for having irrational or inappropriate emotions” (non-judgment). The total score across all item has been frequently used as a global index of dispositional mindfulness[20,21].

Table 2. Deviations from Open Science Framework pre-registration

Some deviations were made from the pre-registered analysis plan in order to streamline analyses, evaluate impact of missingness, account for skewed usage variables, control for multiple comparisons, assess potential adverse effects, and evaluate the impact of influential points on statistical significance. First, due to the amount of missing data, our primary analyses used mixed effects models (MLM)[22] with all three timepoints and maximum likelihood estimation instead of separate analysis of variance (ANOVA) models examining pre-test to mid-treatment and pre-test to post-test. Maximum likelihood can account for data that are missing at random (MAR)[23]. Based on the possibility that missingness was related to the unobserved value itself (i.e., missing not at random [MNAR]), we also conducted sensitivity analyses described below with varying assumptions about the missing data. Second, as our three measures of psychological distress were highly correlated at baseline (*r*s>.70), we created a composite psychological distress measure in order to simplify our analytic plan. Third, our recruited sample size was slightly larger than our pre-registered sample size of 300. Fourth, our measure of therapeutic alliance was not usable due to an error in its administration. Fifth, due to non-normally distributed usage variables, we operationalized usage as a median split of the least skewed/kurtotic variable (days of use). Sixth, we tested the statistical significance of the indirect effects in our mediation models using quasi-Bayesian confidence intervals which is generally preferred to the Baron and Kenny[24] method we initially proposed[25]. Seventh, we applied a Benjmani-Hochberg[26] *p*-value correction to control the false discovery rate (FDR) in each set of analyses. Eighth, we report results for changes in mindfulness. Ninth, we report the proportion of participants showing minimally importance differences in distress (i.e., *d*=0.30)[27] in order to characterize potential harm[28]. Tenth, we evaluated influential points specific for MLMs by assessing change in statistical significance with cases sequentially removed[29].

Table 3. Model formula for primary analyses

$$Y_{ij}=\beta_{00}+ \beta_{10}(Time)+\beta_{20}(Group)+\beta_{30}(Time X Group)+[U_{0_{j}}+e_{ij}]$$

where *Y_ij_* reflects the outcome (e.g., psychological distress) of a given participant (*i*) at a given time (*j*) (i.e., pre-, mid-, or post-test). The fixed intercept (*ß*_00_) reflects the grand mean of the outcome at time = 0 (i.e., pre-test). The fixed effect for time (*ß*_10_) reflects the overall mean linear change in outcome across all participants. The fixed effect for group (*ß*_20_) reflects group differences across all timepoints. Group was coded as either Connection (1) versus Insight (0) or active (1) versus waitlist (0). The fixed effect for time X group (*ß*_30_) reflects the degree to which the linear effect for time varies depending on group status (i.e., whether the trajectory of change differs across groups). The parameters inside the brackets were random effects. Participants’ variability around the fixed intercept was modeled with a random intercept coefficient (*U*_0_*_j_*) indexing participant *j*’s deviation from the overall mean outcome at time = 0 (*ß*_00_). The final component, *e_ij_* reflects the error of prediction or residual for participant *i* at time *j*. Models with random slope coefficients (i.e., allowing trajectories of change to vary not only between groups but between individuals) were not identifiable. This was likely due to the degree of missingness and the limited number of participants with all three observations.

The R syntax for running this model with data in long format (i.e., one row per participant per timepoint per outcome) is:

summary(lmer(value ~ time*group + (1|id), data = df.long[df.long$distress==1,]))

Table 4. Baseline correlations between measures

|  | 1 | 2 | 3 | 4 | 5 | 6 | 7 | 8 | 9 | 10 | 11 |
| --- | --- | --- | --- | --- | --- | --- | --- | --- | --- | --- | --- |
| 1. Depression |  |  |  |  |  |  |  |  |  |  |  |
| 2. Anxiety | .72*** |  |  |  |  |  |  |  |  |  |  |
| 3. Stress | .74*** | .75*** |  |  |  |  |  |  |  |  |  |
| 4. Distress | .90*** | .91*** | .91*** |  |  |  |  |  |  |  |  |
| 5. Social Connect | -.62*** | -.46*** | -.54*** | -.60*** |  |  |  |  |  |  |  |
| 6. Empathy | .08 | .20*** | .12* | .15** | .13* |  |  |  |  |  |  |
| 7. Compassion | -.01 | .08 | .04 | .04 | .20*** | .58*** |  |  |  |  |  |
| 8. Self-Reflection | .00 | .12* | .06 | .07 | .13* | .32*** | .38*** |  |  |  |  |
| 9. Insight | -.47*** | -.42*** | -.46*** | -.50*** | .50*** | .03 | .19*** | .27*** |  |  |  |
| 10. Rumination | .61*** | .66*** | .66*** | .71*** | -.53*** | .19*** | .01 | .10' | -.51*** |  |  |
| 11. Defusion | -.46*** | -.47*** | -.53*** | -.53*** | .46*** | -.06 | .15** | .06 | .42*** | -.56*** |  |
| 12. Mindfulness | -.55*** | -.55*** | -.61*** | -.63*** | .57*** | -.06 | .16** | .13* | .68*** | -.64*** | .63*** |

Note: Depression=PROMIS Depression; Anxiety=PROMIS Anxiety; Stress=Perceived Stress Scale; Psych Distress=composite of PROMIS Depression, PROMIS Anxiety, and Perceived Stress Scale; Social Connect=Social Connectedness Scale; Empathy=Interpersonal Reactivity Index; Compassion=Compassionate Love Scale; Self-Reflection subscale and Insight subscale=subscales of the Self-Reflection and Insight Scale; Rumination=Perseverative Thinking Questionnaire; Defusion=Drexel Defusion Scale; Mindfulness=total score of Five Facet Mindfulness Questionnaire. *n* = 343. Values are Pearson’s *r* correlation coefficient.

**P*<.050; ***P*<.010; ****P*<.001

Table 5. Results of multilevel models assessing differential change over time with outliers removed

|  | CO versus IN | | | | | Active versus WL | | | | | | |
| --- | --- | --- | --- | --- | --- | --- | --- | --- | --- | --- | --- | --- |
| Outcome | *d*_CO_ | *d*_IN_ | *d*_diff_ | *P* | *P*_FDR_ | *d*_active_ | *d*_WL_ | *d*_diff_ | *P* | *P*_FDR_ | Elev *P* | Elev *P*_FDR_ |
| Psychological Distress | -0.78 | -0.70 | -0.08 | .896 | .977 | -0.74 | -0.46 | -0.28 | <.001 | <.001 | .001 | .002 |
| Social Connection | 0.42 | 0.36 | 0.06 | .540 | .972 | 0.39 | 0.16 | 0.23 | .003 | .007 | .012 | .018 |
| Empathy | -0.14 | -0.02 | -0.12 | .374 | .972 | -0.08 | -0.13 | 0.05 | .623 | .623 | .431 | .431 |
| Compassion | 0.11 | 0.34 | -0.23 | .290 | .972 | 0.22 | 0.11 | 0.11 | .146 | .164 | .191 | .215 |
| Self-Reflection subscale | 0.08 | 0.18 | -0.10 | .663 | .977 | 0.13 | 0.04 | 0.09 | .017 | .022 | .040 | .051 |
| Insight subscale | 0.46 | 0.39 | 0.07 | .977 | .977 | 0.42 | 0.29 | 0.13 | .014 | .021 | .001 | .002 |
| Rumination | -0.45 | -0.56 | 0.11 | .321 | .972 | -0.5 | -0.32 | -0.18 | .010 | .018 | .007 | .013 |
| Defusion | 0.83 | 0.66 | 0.17 | .780 | .977 | 0.75 | 0.38 | 0.37 | <.001 | <.001 | <.001 | <.001 |
| Mindfulness | 0.93 | 0.70 | 0.23 | .514 | .972 | 0.80 | 0.46 | 0.34 | <.001 | <.001 | <.001 | <.001 |

Note: CO=Awareness + Connection; IN=Awareness + Insight; Active=combined Awareness + Connection and Awareness + Insight; WL=waitlist; Psych Distress=composite of PROMIS Depression, PROMIS Anxiety, and Perceived Stress Scale; Social Connection=Social Connectedness Scale; Empathy=Interpersonal Reactivity Index; Compassion=Compassionate Love Scale; Self-Reflection subscale and Insight subscale=subscales of the Self-Reflection and Insight Scale; Rumination=Perseverative Thinking Questionnaire; Defusion=Drexel Defusion Scale; Mindfulness=total score of Five Facet Mindfulness Questionnaire; *d=*Cohen’s *d*, calculated as pre-post for within-group effects and the difference between within-group effects (Connection minus Insight, active minus waitlist) for *d*_diff_; for within-group; *p=p-*value from time X group interaction from multilevel models; FDR=False-Discovery Rate-adjusted *p-*values; Elev=active versus waitlist time X group interaction restricted to sample with elevated depression and/or anxiety at baseline (T ≥ 55). Outlier defined as three standard deviations above or below the mean.

Table 6. Wilcoxon rank sum test using the completer sample and a worst-case scenario assumption for missingness

|  | Completer sample | | | | Worst-case scenario | | | |
| --- | --- | --- | --- | --- | --- | --- | --- | --- |
| Outcome | Rank_active_ | Rank_WL_ | *P* | *P*_FDR_ | Rank_active_ | Rank_WL_ | *P* | *P*_FDR_ |
| Psychological Distress | 69.11 | 93.61 | .001 | .003 | 178.10 | 159.90 | .080 | .158 |
| Social Connection | 85.65 | 64.64 | .004 | .009 | 166.91 | 182.08 | .142 | .168 |
| Empathy | 80.36 | 73.37 | .339 | .339 | 164.48 | 186.91 | .030 | .158 |
| Compassion | 82.33 | 69.38 | .075 | .085 | 165.61 | 184.68 | .065 | .158 |
| Self-Reflection subscale | 84.09 | 66.87 | .018 | .033 | 165.88 | 184.13 | .077 | .158 |
| Insight subscale | 83.58 | 67.60 | .028 | .042 | 166.10 | 183.70 | .088 | .158 |
| Rumination | 70.30 | 85.50 | .037 | .047 | 177.55 | 160.99 | .108 | .162 |
| Defusion | 88.42 | 63.19 | .001 | .003 | 167.00 | 181.92 | .149 | .168 |
| Mindfulness | 91.28 | 60.61 | <.001 | <.001 | 167.85 | 180.23 | .233 | .233 |

Note: Rank=mean pre-post residual rank; Active=combined Awareness + Connection and Awareness + Insight; WL=waitlist; Psychological Distress=composite of PROMIS Depression, PROMIS Anxiety, and Perceived Stress Scale; Social Connection=Social Connectedness Scale; Empathy=Interpersonal Reactivity Index; Compassion=Compassionate Love Scale; Self-Reflection subscale and Insight subscale=subscales of the Self-Reflection and Insight Scale; Rumination=Perseverative Thinking Questionnaire; Defusion=Drexel Defusion Scale; Mindfulness=total score of Five Facet Mindfulness Questionnaire; *P=P-*value from two-sample Wilcoxon rank sum test (i.e., Mann-Whitney); FDR=False-Discovery Rate-adjusted *P-*values. Worst-case scenario replaced missing values with the maximum or minimum residual, depending on whether a larger or smaller residual indicates improvement. For outcomes in which lower scores are better (e.g., psychological distress), lower mean rank indicates larger reductions. For outcomes in which higher scores are better (e.g., social connectedness), high mean rank indicates larger increases.

Table 7. Wilcoxon rank sum test results for three missingness assumptions

|  | 0.25 *SD* | | | | 0.50 *SD* | | | | 0.75 *SD* | | | |
| --- | --- | --- | --- | --- | --- | --- | --- | --- | --- | --- | --- | --- |
| Outcome | Rank_active_ | Rank_WL_ | *P* | *P*_FDR_ | Rank_active_ | Rank_WL_ | *P* | *P*_FDR_ | Rank_active_ | Rank_WL_ | *P* | *P*_FDR_ |
| Psychological Distress | 163.06 | 189.73 | .010 | .031 | 164.98 | 185.92 | .044 | .133 | 167.32 | 181.28 | .180 | .541 |
| Social Connection | 180.20 | 155.74 | .018 | .040 | 174.91 | 166.23 | .401 | .600 | 173.71 | 168.61 | .622 | .912 |
| Empathy | 174.18 | 167.68 | .530 | .530 | 172.11 | 171.78 | .975 | .975 | 171.61 | 172.77 | .912 | .912 |
| Compassion | 177.83 | 160.44 | .093 | .139 | 171.63 | 172.74 | .915 | .975 | 171.34 | 173.31 | .849 | .912 |
| Self-Reflection subscale | 176.93 | 162.22 | .155 | .179 | 174.52 | 167.00 | .467 | .600 | 172.64 | 170.74 | .855 | .912 |
| Insight subscale | 176.88 | 162.32 | .159 | .179 | 175.68 | 164.7 | .288 | .600 | 170.77 | 174.44 | .722 | .912 |
| Rumination | 165.69 | 184.50 | .068 | .123 | 169.23 | 177.49 | .424 | .600 | 173.19 | 169.63 | .731 | .912 |
| Defusion | 181.72 | 152.73 | .005 | .023 | 179.75 | 156.63 | .026 | .116 | 176.89 | 162.31 | .160 | .541 |
| Mindfulness | 185.45 | 145.34 | <.001 | .001 | 181.57 | 153.02 | .006 | .054 | 179.94 | 156.26 | .023 | .203 |

Note: 0.25 *SD*, 0.50 *SD*, 0.75 *SD*=missing values assumed to be 0.25, 0.50, or .075 standard deviation above or below the mean residual, depending on whether larger or smaller residuals indicates improvement; Rank=mean pre-post residual rank; Active=combined Awareness + Connection and Awareness + Insight; WL=waitlist; Psychological Distress=composite of PROMIS Depression, PROMIS Anxiety, and Perceived Stress Scale; Social Connection=Social Connectedness Scale; Empathy=Interpersonal Reactivity Index; Compassion=Compassionate Love Scale; Self-Reflection subscale and Insight subscale=subscales of the Self-Reflection and Insight Scale; Rumination=Perseverative Thinking Questionnaire; Defusion=Drexel Defusion Scale; Mindfulness=total score of Five Facet Mindfulness Questionnaire; *P=P-*value from two-sample Wilcoxon rank sum test (i.e., Mann-Whitney); FDR=False-Discovery Rate-adjusted *P-*values. For outcomes in which lower scores are better (e.g., psychological distress), lower mean rank indicates larger reductions. For outcomes in which higher scores are better (e.g., social connectedness), high mean rank indicates larger increases.

Figure 1. Histograms displaying distribution of usage variables. days=days of use; practice=number of meditation practice sessions completed; activities=number of total activities completed (meditation practices sessions and didactic content); mins=minutes of meditation practice.

Figure 2. Survival curves for Awareness + Connection (CO) and Awareness + Insight (IN) participants. with 95% confidence intervals. Usage calculated from completion of pre-test survey to 9 weeks (63 days). Groups did not differ in engagement over time (hazard ratio=1.17, *P*=.242, with Insight as reference group).

Figure 3. Baseline variables moderate longitudinal changes in psychological distress. Participants higher in baseline rumination and empathy and lower in baseline defusion showed relatively larger reductions in distress.

**References**

1. Pilkonis PA, Choi SW, Reise SP, Stover AM, Riley WT, Cella D, PROMIS Cooperative Group. Item banks for measuring emotional distress from the Patient-Reported Outcomes Measurement Information System (PROMIS®): depression, anxiety, and anger. Assessment 2011 Sep;18(3):263–283. PMID:21697139

2. Choi SW, Schalet B, Cook KF, Cella D. Establishing a common metric for depressive symptoms: Linking the BDI-II, CES-D, and PHQ-9 to PROMIS depression. Psychol Assess 2014 Jun;26(2):513–527. PMID:24548149

3. Schalet BD, Cook KF, Choi SW, Cella D. Establishing a common metric for self-reported anxiety: Linking the MASQ, PANAS, and GAD-7 to PROMIS Anxiety. J Anxiety Disord 2014 Jan;28(1):88–96. PMID:24508596

4. Cohen S, Williamson GM. Perceived stress in a probability sample of the United States. In: Spacapan S, Oskamp S, editors. Soc Psychol Health Claremont Symp Appl Soc Psychol Newbury Park, CA: Sage; 1988. p. 31–67.

5. Roberti JW, Harrington LN, Storch EA. Further psychometric support for the 10‐item version of the Perceived Stress Scale. J Coll Couns 2006;9(2):135–147. [doi: 10.1002/j.2161-1882.2006.tb00100.x]

6. Lee RM, Draper M, Lee S. Social connectedness, dysfunctional interpersonal behaviors, and psychological distress: Testing a mediator model. J Couns Psychol US: American Psychological Association; 2001;48(3):310–318. [doi: 10.1037/0022-0167.48.3.310]

7. Lee RM, Dean BL, Jung K-R. Social connectedness, extraversion, and subjective well-being: Testing a mediation model. Personal Individ Differ 2008 Oct;45(5):414–419. [doi: 10.1016/j.paid.2008.05.017]

8. Cordier R, Milbourn B, Martin R, Buchanan A, Chung D, Speyer R. A systematic review evaluating the psychometric properties of measures of social inclusion. PloS One 2017;12(6):e0179109. PMID:28598984

9. Lang AJ, Malaktaris AL, Casmar P, Baca SA, Golshan S, Harrison T, Negi L. Compassion meditation for posttraumatic stress disorder in veterans: A randomized proof of concept study. J Trauma Stress 2019 Apr;32(2):299–309. PMID:30929283

10. Davis MH. Measuring individual differences in empathy: Evidence for a multidimensional approach. J Pers Soc Psychol US: American Psychological Association; 1983;44(1):113–126. [doi: 10.1037/0022-3514.44.1.113]

11. Cliffordson C. The hierarchical structure of empathy: Dimensional organization and relations to social functioning. Scand J Psychol 2002 Feb;43(1):49–59. PMID:11885760

12. Hojat M, Mangione S, Kane GC, Gonnella JS. Relationships between scores of the Jefferson Scale of Physician Empathy (JSPE) and the Interpersonal Reactivity Index (IRI). Med Teach 2005 Nov;27(7):625–628. PMID:16332555

13. Sprecher S, Fehr B. Compassionate love for close others and humanity. J Soc Pers Relatsh SAGE Publications Ltd; 2005 Oct;22(5):629–651. [doi: 10.1177/0265407505056439]

14. Grant AM, Franklin J, Langford P. The self-reflection and insight scale: A new measure of private self-consciousness. Soc Behav Personal Int J New Zealand: Society for Personality Research; 2002;30(8):821–835. [doi: 10.2224/sbp.2002.30.8.821]

15. Harrington R, Loffredo DA, Perz CA. Dispositional mindfulness as a positive predictor of psychological well-being and the role of the private self-consciousness insight factor. Personal Individ Differ 2014 Dec;71:15–18. [doi: 10.1016/j.paid.2014.06.050]

16. Ehring T, Zetsche U, Weidacker K, Wahl K, Schönfeld S, Ehlers A. The Perseverative Thinking Questionnaire (PTQ): Validation of a content-independent measure of repetitive negative thinking. J Behav Ther Exp Psychiatry 2011 Jun;42(2):225–232. PMID:21315886

17. Forman EM, Herbert JD, Juarascio AS, Yeomans PD, Zebell JA, Goetter EM, Moitra E. The Drexel Defusion Scale: A new measure of experiential distancing. J Context Behav Sci 2012 Dec;1(1–2):55–65. [doi: 10.1016/j.jcbs.2012.09.001]

18. Van Dam NT, Earleywine M, Danoff-Burg S. Differential item function across meditators and non-meditators on the Five Facet Mindfulness Questionnaire. Personal Individ Differ 2009 Oct;47(5):516–521. [doi: 10.1016/j.paid.2009.05.005]

19. Baer RA, Smith GT, Hopkins J, Krietemeyer J, Toney L. Using self-report assessment methods to explore facets of mindfulness. Assessment 2006 Mar;13(1):27–45. PMID:16443717

20. Goldberg SB, Del Re AC, Hoyt WT, Davis JM. The secret ingredient in mindfulness interventions? A case for practice quality over quantity. J Couns Psychol 2014 Jul;61(3):491–497. PMID:25019551

21. Lykins ELB, Baer RA. Psychological functioning in a sample of long-term practitioners of mindfulness meditation. J Cogn Psychother 2009;23(3):226–241. [doi: 10.1891/0889-8391.23.3.226]

22. Snijders TAB, Bosker RJ. Multilevel analysis: An introduction to basic and advance multilevel modeling. 2nd ed. London: Sage; 2012.

23. Graham JW. Missing data analysis: Making it work in the real world. Annu Rev Psychol 2009;60:549–576. PMID:18652544

24. Baron RM, Kenny DA. The moderator-mediator variable distinction in social psychological research: conceptual, strategic, and statistical considerations. J Pers Soc Psychol 1986 Dec;51(6):1173–1182. PMID:3806354

25. Imai K, Keele L, Tingley D. A general approach to causal mediation analysis. Psychol Methods 2010 Dec;15(4):309–334. PMID:20954780

26. Benjamini Y, Hochberg Y. Controlling the false discovery rate: A practical and powerful approach to multiple testing. J R Stat Soc Ser B Methodol [Royal Statistical Society, Wiley]; 1995;57(1):289–300. [doi: 10.2307/2346101]

27. Schalet BD, Pilkonis PA, Yu L, Dodds N, Johnston KL, Yount S, Riley W, Cella D. Clinical validity of PROMIS Depression, Anxiety, and Anger across diverse clinical samples. J Clin Epidemiol 2016 May;73:119–127. PMID:26931289

28. Hirshberg MJ, Goldberg SB, Rosenkranz M, Davidson RJ. Prevalence of harm in mindfulness-based stress reduction. Psychol Med [Internet] in press; PMID:32807249

29. Nieuwenhuis R, Grotenhuis M te, Pelzer B. Influence. ME: Tools for detecting influential data in mixed effects models. R J 2012;4(2):38–47. [doi: 10.32614/RJ-2012-011]
